# Whence Feral Vaccinia?

**DOI:** 10.3201/eid1606.100315

**Published:** 2010-06

**Authors:** Richard C. Condit

**Affiliations:** University of Florida, Gainesville, Florida, USA

**Keywords:** Smallpox, vaccinia, poxvirus, viruses, commentary

When the World Health Organization declared smallpox eradicated in 1979, smallpox vaccination was discontinued worldwide. Although cessation of smallpox vaccination is well justified, given the risks associated with complications from the vaccine, lack of vaccination nevertheless creates a growing population of persons now susceptible to infection by a few poxviruses previously covered by the smallpox vaccine. These include the orthopoxviruses monkeypox; cowpox; and, ironically, vaccinia, the virus used for smallpox vaccination. Although few persons die from these infections, they are nevertheless a public health nuisance and expense. Thus, understanding the epidemiology of these viruses is in the interest of public health.

The most common vaccine-preventable poxvirus infections in humans are cowpox and monkeypox (*1*). Both are zoonoses; the natural host for each seems to be rodents, and transmission occurs through close contact. Monkeypox virus occurs in western and central Africa, causes a disseminated infection in humans, can be transmitted among humans at a low rate, and is associated with a 1%–10% (depending on the virus strain) case-fatality rate. Cowpox virus is relatively common in Europe and Asia, causes a limited exanthema, and does not often cause death of previously healthy persons.

Vaccinia, the virus used in the live smallpox vaccine, was originally isolated in the late 18th century from persons with illness that clinically resembled cowpox. However, modern genomics have shown that the vaccinia virus strains used for smallpox control in the 20th and 21st centuries are genetically distinct from cowpox viruses currently circulating. In fact, with 2 notable exceptions, vaccinia virus is not found in nature. However, vaccinia infection has been documented in India and Brazil (*2*). In India, some strains of buffalopox, transmitted to humans through buffaloes, appear to be vaccinia. Likewise, in Brazil, reports of a cowpox-like disease, caused by a vaccinia virus and transmitted from cattle to humans, have increased substantially since the first report in 2000. In both outbreaks (India and Brazil), evidence suggests that the original source of the virus was the smallpox vaccine virus that has been introduced into the wild—or feral vaccinia, as it has sometimes been called. The reservoirs for these viruses in the wild are not well understood.

In this issue of Emerging Infectious Diseases, Abrahão et al. (*3*) describe a serosurvey for orthopoxvirus among 344 wild animals from the Brazilian Amazonia ecosystem. The animals, 296 monkeys and a variety of other mammals, were captured by a fauna-rescue program during the construction of a hydroelectric plant in Tocantins State, Brazil, far removed from other human activity. Of these animals, 84 (24%), predominantly monkeys, were seropositive for orthopoxvirus. Furthermore, 18 serum samples were positive for orthopoxvirus DNA according to PCR. From these 18 positive samples, sequencing of 6 isolates revealed the vaccinia strain commonly associated with vaccinia outbreaks among cattle and humans in Brazil.

These finding suggest a remarkably high incidence of vaccinia infection in the Brazilian wilderness and in a host, namely monkeys, not normally considered as an active reservoir for orthopoxviruses. Although Abrahão et al. do not specifically identify monkeys as a primary reservoir for vaccinia virus and do not address the mode of transmission of the virus among these animals, the results suggest a substantial repository of vaccinia virus in the Brazilian wilderness. Especially given the broad host range of vaccinia, these findings warrant a substantial effort to characterize further the circulation of vaccinia virus in this region. If the results described in the article by Abrahão et al. (*3*) can be confirmed and expanded by other laboratories, they would have major implications for public health.
